# Improved protein splicing through viral passaging

**DOI:** 10.1128/mbio.00984-24

**Published:** 2024-05-23

**Authors:** Adam J. Hume, Dylan J. Deeney, John S. Smetana, Jacquelyn Turcinovic, John H. Connor, Marlene Belfort, Elke Mühlberger, Christopher W. Lennon

**Affiliations:** 1 Department of Microbiology, Boston University School of Medicine, Boston, Massachusetts, USA; 2 National Emerging Infectious Diseases Laboratories, Boston University, Boston, Massachusetts, USA; 3 Center for Emerging Infectious Diseases Policy & Research, Boston University, Boston, Massachusetts, USA; 4 Department of Biological Sciences, Murray State University, Murray, Kentucky, USA; 5 Department of Biological Sciences and RNA Institute, University at Albany, Albany, New York, USA; UT Southwestern Medical Center, Dallas, Texas, USA

**Keywords:** protein splicing, Ebola virus, intein, self-splicing fluorescent reporter, reporter virus

## Abstract

Intervening proteins (inteins) are translated as subdomains within host proteins and removed through an intein-driven splicing reaction where the flanking sequences (exteins) are joined with a peptide bond. Previously, we developed a self-removing translation reporter for labeling Ebola virus (EBOV). In this reporter, an intein (RadA) containing the fluorescent protein ZsGreen (ZsG) is inserted within the EBOV protein VP30. Upon VP30-RadA-ZsG expression from the viral genome, RadA-ZsG is removed from VP30 through the protein splicing activity of RadA, generating functional, non-tagged VP30 and functional ZsGreen. While incorporation of our VP30-RadA-ZsG fusion reporter into recombinant EBOV (rEBOV-RadA-ZsG) resulted in an infectious virus that expresses ZsG upon infection of cells, this virus displayed a replication defect compared to wild-type EBOV, which might be the result of insufficient RadA splicing. Here, we demonstrate that the serial passaging of rEBOV-RadA-ZsG in human cells led to an increase in replication efficiency compared to unpassaged rEBOV-RadA-ZsG. Sequencing of passaged viruses revealed intein-specific mutations. These mutations improve intein activity in both prokaryotic and eukaryotic systems, as well as in multiple extein contexts. Taken together, our findings offer a novel means to select for inteins with enhanced catalytic properties that appear independent of extein context and expression system.

**IMPORTANCE:**

Intervening proteins (inteins) are self-removing protein elements that have been utilized to develop a variety of innovative protein engineering technologies. Here, we report the isolation of inteins with improved catalytic activity through viral passaging. Specifically, we inserted a highly active intein within an essential protein of Ebola virus and serially passaged this recombinant virus, which led to intein-specific hyper-activity mutations. The identified mutations showed improved intein activity within both bacterial and eukaryotic expression systems and in multiple extein contexts. These results present a new strategy for developing inteins with improved splicing activity.

## OBSERVATION

Inteins (intervening proteins) are translated within host proteins and removed through protein splicing (1, 2). During the intein-catalyzed protein splicing reaction, the intein is removed and the flanking sequences, known as N- and C-exteins, are joined with a peptide bond. The ability to precisely rearrange peptide bonds in a controlled manner has led to a variety of innovative technologies including, but not limited to, methods for bioseparations, assembly of semisynthetic proteins, and transgene delivery (2, 3).

**Fig 1:**
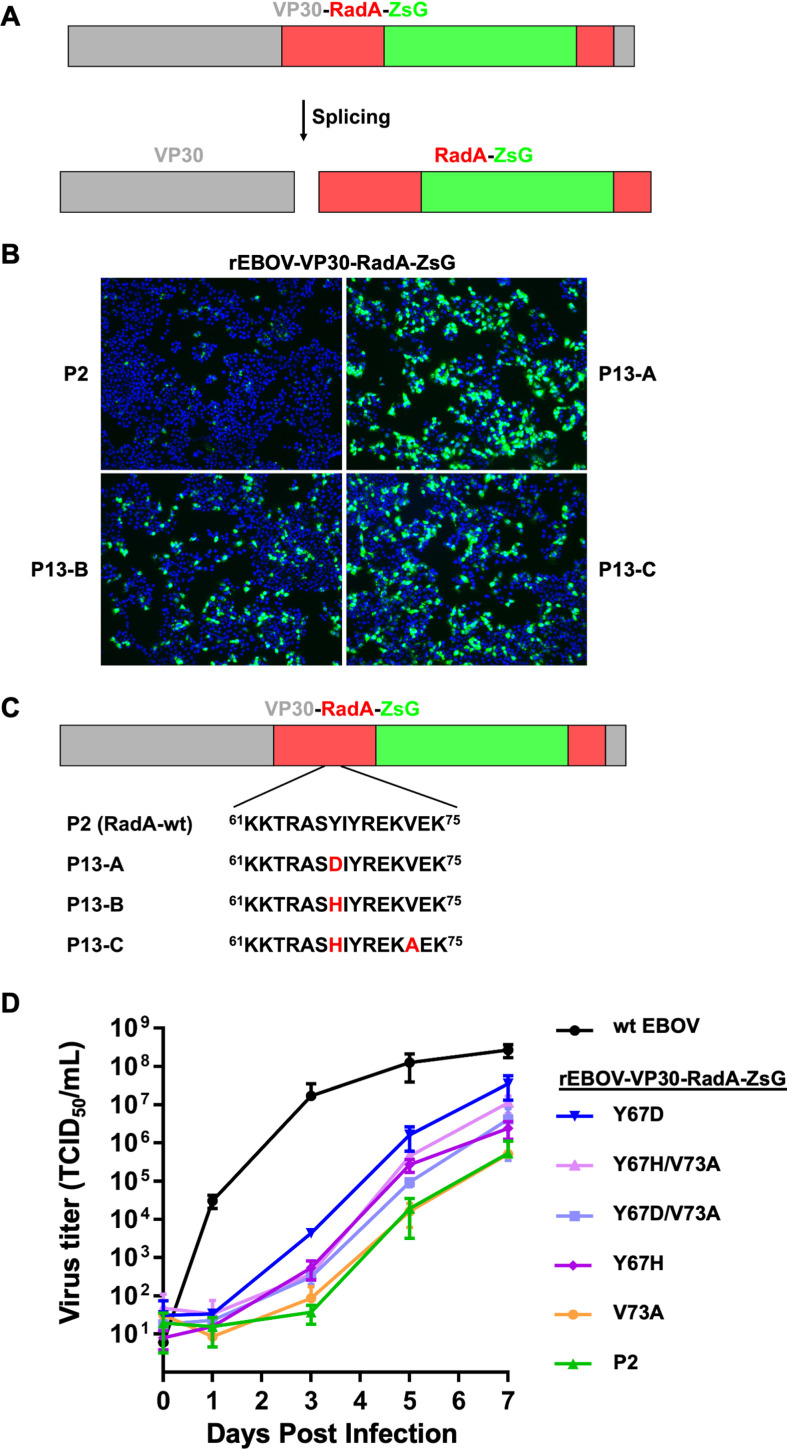
Passaging of rEBOV expressing VP30-RadA-ZsG reporter results in intein-specific mutations that improve viral replication. (A) Schematic of VP30-RadA-ZsG. (B) rEBOV-VP30-RadA-ZsG was serially passaged in Huh7 cells. Passage 2 (**P2**) and three independent lineages of serially passaged rEBOV-VP30-RadA-ZsG (P13A-C) were used to infect Huh7 cells at an MOI of 1. Live cell fluorescent images of Hoechst-stained cells were taken 1 dpi. (C) Sequencing of the three P13 lineages revealed mutations at RadA position 67 in all the three (Y67D or Y67H). Lineage C had had an additional V73A mutation. Mutations are shown in red. (D) Growth curves of rEBOV expressing wt (P2) and variant intein-ZsG reporters as indicated. In panels A and D, VP30 is colored gray, the intein is colored red, and ZsG is colored green. The bars are drawn to scale.

Ebola virus (EBOV) belongs to the group of nonsegmented negative-sense RNA viruses and causes a serious disease in humans with case fatality rates ranging from 40% to 90% (4). We recently developed recombinant EBOV containing an intein-based fluorescent reporter. This self-removing reporter system consists of the fluorescent protein ZsGreen (ZsG) inserted into the *Pyrococcus horikoshii* RadA intein (RadA). This reporter (RadA-ZsG) is both splicing-active and fluorescent. The RadA-ZsG fusion was inserted within the gene encoding the EBOV transcriptional activator VP30, to generate the recombinant virus rEBOV-VP30-RadA-ZsG (Fig. 1A) (5). We previously reported that this rEBOV reporter virus was both fluorescent and infectious but had a replication defect compared to wild-type virus (5).

We reasoned that serial passaging of our initial reporter virus in mammalian cells could select for adaptive mutations that compensate for the rEBOV-VP30-RadA-ZsG replication defect. Using this strategy, we passaged three independent lineages of rEBOV-VP30-RadA-ZsG, a total of 13 times in the human immortalized liver carcinoma cell line Huh7. In each lineage, passage 13 (P13) virus showed higher infection rates compared to the passage 2 (P2) virus as shown by ZsG fluorescence (Fig. 1B).

Sequence analysis revealed that each virus lineage had accumulated mutations during passaging. Intriguingly, this included missense mutations that mapped to intein codons. Specifically, all three lineages had a missense mutation within RadA codon 67 (Y67D or Y67H), with one lineage also having a secondary mutation within codon 73 (V73A) in addition to Y67H (Fig. 1C). While additional mutations were observed elsewhere in the genome (Table S1), including additional missense mutations within RadA and ZsG for one of the three lineages (Table S1; P13-B), we chose to focus on codons 67 and 73 since we observed mutations of codon 67 in all three lineages and because codon 73 was previously observed to make contacts to the −1 residue near the intein active site (6).

We next determined if any of the three RadA mutations at position Y67D, Y67H, or V73A could rescue the replication defect observed in our initial reporter virus. To deconvolute the effect of these mutations of interest from other mutations within the genome of the passaged viruses, we generated five different rEBOV-VP30-RadA-ZsG viruses with single (Y67D, Y67H, or V73A) or double substitutions (Y67D/V73A and Y67H/V73A) within RadA and compared their replication kinetics to a low passage (P2) rEBOV-VP30-RadA-ZsG in Huh7 cells. Excitingly, these RadA mutations each led to increased viral replication compared to the P2 rEBOV-VP30-RadA-ZsG with the exception of the V73A mutation (Fig. 1D). In particular, the virus containing the Y67D single substitution replicated to titers several orders of magnitude higher than the low passage, wild-type RadA-containing virus (Fig. 1D). While a substantial increase in viral propagation was observed for several mutants, these viruses still exhibited a replication defect compared to wild-type EBOV (Fig. 1D).

**Fig 2:**
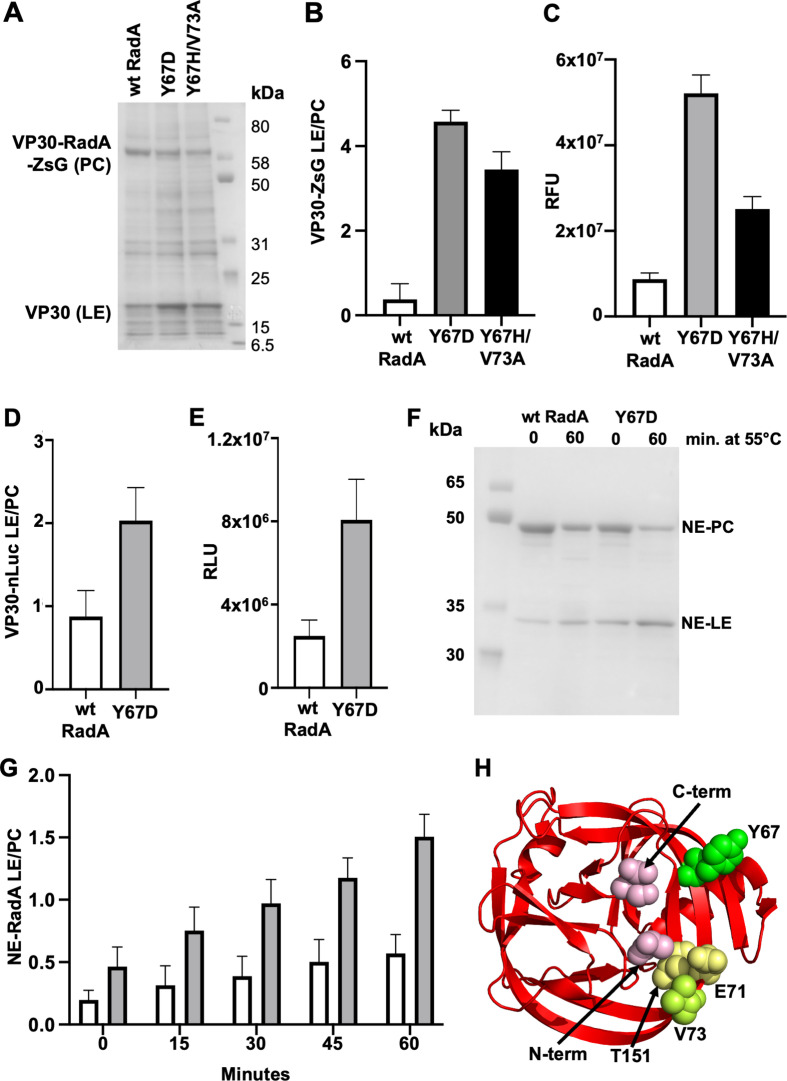
Intein mutations identified in serially passaged rEBOV-VP30-RadA-ZsG generally improve intein activity. (A) SDS-PAGE following expression and quantitative pull-down of VP30-RadA-ZsG (wild-type and mutants) from whole cell lysates. The expected sizes are 18.6 kDa for the LE and 64.5 kDa for the PC. (B) Quantification of the ratio of VP30 (LE) levels to VP30-RadA-ZsG precursor (PC) from panel A. (C) Relative fluorescence units (RFU) from the soluble lysate VP30-RadA-ZsG (wild type and mutants). (D)Quantification of the ratio of VP30 (LE) levels to the VP30-RadA-nLuc precursor (PC). (E) Relative luciferase units (RLU) from cell lysates used for pull-downs in panel D. (F) SDS-PAGE of RadA within its native extein (NE) context following purification (0 min) and incubation at 55°C for 60 min. PC, precursor; LE, ligated exteins. (G) Splicing of RadA NE following incubation at 55°C for indicated times. (H) RadA intein structure (PDB 4E2T) with various residues of interest in space fill and labeled. In panels B, C, E, F, and H, quantification is from three independent experiments. Error bars are based on standard deviation from three independent experiments. VP30 has residues 1–141 deleted to improve expression in *E. coli*.

To test if these intein mutations could improve VP30-RadA-ZsG splicing outside of the context of the virus, we tested the mutations using an *E. coli* VP30 expression construct. It was previously reported that VP30 expresses poorly in *E. coli*, but that deletion of residues 1–141 improves expression (7). We therefore created a truncated *E. coli* VP30 expression construct spanning amino acids 142–288, with RadA-ZsG inserted between residues 262–263 of VP30 and an N-terminal His-tag (Fig. S1A), which can be used to isolate both unspliced VP30-RadA-ZsG and spliced VP30. Following expression in *E. coli*, quantitative His-tag pull-downs were performed on soluble lysates and analyzed by SDS-PAGE. We chose to focus on the Y67D and Y67H/V73A substitutions as they exhibited the least replication defect compared to wild-type EBOV (Fig. 1D). Remarkably, the introduction of the Y67D substitution into the *E. coli* VP30-RadA-ZsG expression construct resulted in an almost 5-fold increase in total soluble spliced VP30 compared to wild-type RadA (Fig. S1A through C). The Y67H/V73A double substitution in RadA led to a 2-fold increase in VP30 (Fig. S1A through C). While no VP30-RadA-ZsG precursor was observed following pull-down in soluble lysates (Fig. S1B), both precursor and ligated exteins were isolated from the whole cell fraction (Fig. 2A), which contains both soluble and insoluble proteins. Comparison of the whole cell fraction also indicated more splicing for the Y67D and Y67H/V73A, with a greater than 5-fold increase in the ratio of ligated exteins to precursor compared to wild-type for both variants (Fig. 2B). Importantly, the appearance of precursor unequivocally attributed mutant phenotypes to enhanced protein splicing. Additionally, expression levels of the wild-type and mutant constructs were similar, eliminating the possibility that differences observed were due to variable expression levels. We also reasoned that, if mutants Y67D and Y67H/V73A were more efficiently spliced, higher fluorescence measurements should occur in *E. coli* soluble lysates due to increased levels of spliced RadA-ZsG. Consistent with improved splicing, relative fluorescence was more than 6-fold higher for Y67D and 3-fold higher for Y67H/V73A compared to wild-type RadA lysates (Fig. 2C). The increased fluorescence must be a result of increased levels of spliced RadA-ZsG, as no precursor is observed in the soluble fraction (Fig. S1B).

Next, we investigated whether the increased intein catalytic activity was specific for the VP30-RadA-ZsG fusion or a general enhancement of the RadA intein splicing activity. As a first step to test this, we chose to insert another reporter within the RadA intein. We swapped NanoLuc (nLuc) in place of ZsG in the same His-tagged, truncated VP30 *E. coli* expression construct (VP30-RadA-nLuc; Fig. S2A). We chose to focus on the Y67D variant as it provided the largest increase in RadA splicing, VP30 production, and fluorescence for the VP30-RadA-ZsG construct (Fig. 2A through C; Fig. S1) and the best replication kinetics of any mutant rEBOV-VP30-RadA-ZsG (Fig. 1D). Following expression, the Y67D version of VP30-RadA-nLuc showed a greater than 2-fold increase in the relative levels of VP30 compared to the wild-type RadA version (Fig. S2B), as well as a higher ratio of VP30 to unspliced precursor (Fig. 2D). We also measured nLuc activity present in soluble lysates and found a nearly 3-fold higher activity for Y67D compared to wild-type RadA (Fig. 2E), consistent with improved catalytic activity for this intein variant. While this increase in catalytic activity of Y67D was not as high as that of VP30-RadA-ZsG, our results demonstrate an increase independent of the construct that was originally used for viral passaging. Additionally, these results demonstrate that the RadA intein can accommodate nLuc insertion, with the intein and nLuc both retaining activity, consistent with recent work by Truong et al*.* (8).

While substitutions that improve intein activity in a specific extein context (e.g. VP30) are valuable, improvements that are independent of the extein context are more broadly applicable. To determine if the Y67D substitution could improve RadA intein catalysis within another extein context, we examined splicing activity within the native exteins from *P. horikoshii* (NE-RadA). It was shown previously that NE-RadA splicing was largely blocked during expression in *E. coli*, but that splicing could be activated by either elevated temperature or the addition of single-stranded DNA (9–11). Wild-type and Y67D versions of the NE-RadA construct (Fig. S3A) were grown in *E. coli* at 14°C to allow for expression but reduced splicing activity. Following purification, we found that splicing activity of the Y67D NE-RadA mutant was increased more than 2-fold compared to wild-type NE-RadA, indicating a greater intein activity (Fig. 2F and 0 min). Upon incubating these purified lysates at 55°C, which activates the intein, we similarly observed that NE-RadA Y67D was spliced approximately 2-fold faster than the wild-type (Fig. 2F through G; Fig. S3B). Importantly, these data indicate that these mutations enhance RadA splicing in an extein context different from which they were isolated.

Here, we report the discovery of variants of rEBOV-VP30-RadA-ZsG with improved replication efficiency compared to the initial reporter virus isolated through passaging. Sequencing these passaged viruses revealed intein-specific mutations that generally improve the RadA intein activity within multiple contexts and in both mammalian and bacterial systems. Our work not only resulted in the generation of rEBOV reporter viruses with significantly increased replication kinetics but perhaps more importantly, it describes a novel selection engine to generate inteins with enhanced splicing efficiency.

To develop recombinant EBOV containing a self-removing reporter, we focused on the RadA intein because of its naturally high splicing activity. The RadA intein originally gained interest as it was found to be the most active of 20 inteins assayed (12). Additional work has characterized this intein extensively both biochemically and structurally and has shown that it splices efficiently in numerous extein contexts (6, 9–12). Therefore, our approach appears able to improve an intein that already has high activity.

Inteins are members of the HINT (Hog/INTein) auto-processing domain superfamily and have a characteristic horseshoe-like fold that brings the N- and C-terminus into proximity for splicing (Fig. 2H). In a structure of the RadA intein, interactions were observed between the N-extein residue immediately preceding the intein (−1 position) and intein residues E71, V73, and T151 that were proposed to promote productive splicing (12; Fig. 2H). While the mechanism by which the substitutions to Y67 and V73 improve RadA intein activity is unknown, it is intriguing that they are near the N- and C-termini, at the active site of the intein (Fig. 2H).

While approaches for improving inteins in microbial systems exist (13–18), our methodology of serially passaging a virus that contains an intein within an essential viral gene is a novel approach to generate inteins with enhanced splicing efficiency in mammalian cells. Additionally, our results suggest that this approach can lead to a general improvement of intein activity that is independent of extein context or expression system. These data therefore provide a blueprint by which other inteins could be improved via virus passaging-based directed evolution, potentially leading to the enhancement of many intein-based technologies.

## SUPPLEMENTAL MATERIAL

Fig. S1 mbio.00984-24-s0001.tiff Intein substitutions Y67D and Y67H/V73A improve intein activity within VP30-Int-ZsG context.

Fig. S2 mbio.00984-24-s0002.tiff Intein substitution Y67D improves intein activity within VP30-RadA-nLuc context.

Fig. S3 mbio.00984-24-s0003.tiff Intein substitution Y67D improves intein activity within native RadA extein context.

Supplemental Information mbio.00984-24-s0004.docx Supplemental text, tables, and figures.

Table S1 mbio.00984-24-s0005.xlsx Mutations found in passaged rEBOV-VP30-RadA-ZsG lineages.

ASM does not own the copyrights to Supplemental Material that may be linked to, or accessed through, an article. The authors have granted ASM a non-exclusive, world-wide license to publish the Supplemental Material files. Please contact the corresponding author directly for reuse.
