# Supplementary figures and images for: Improved protein splicing through viral passaging
Source: mBio. 2024 May 23;15(6):e00984-24. doi: 10.1128/mbio.00984-24 (PMC11237716; doi:10.1128/mbio.00984-24)

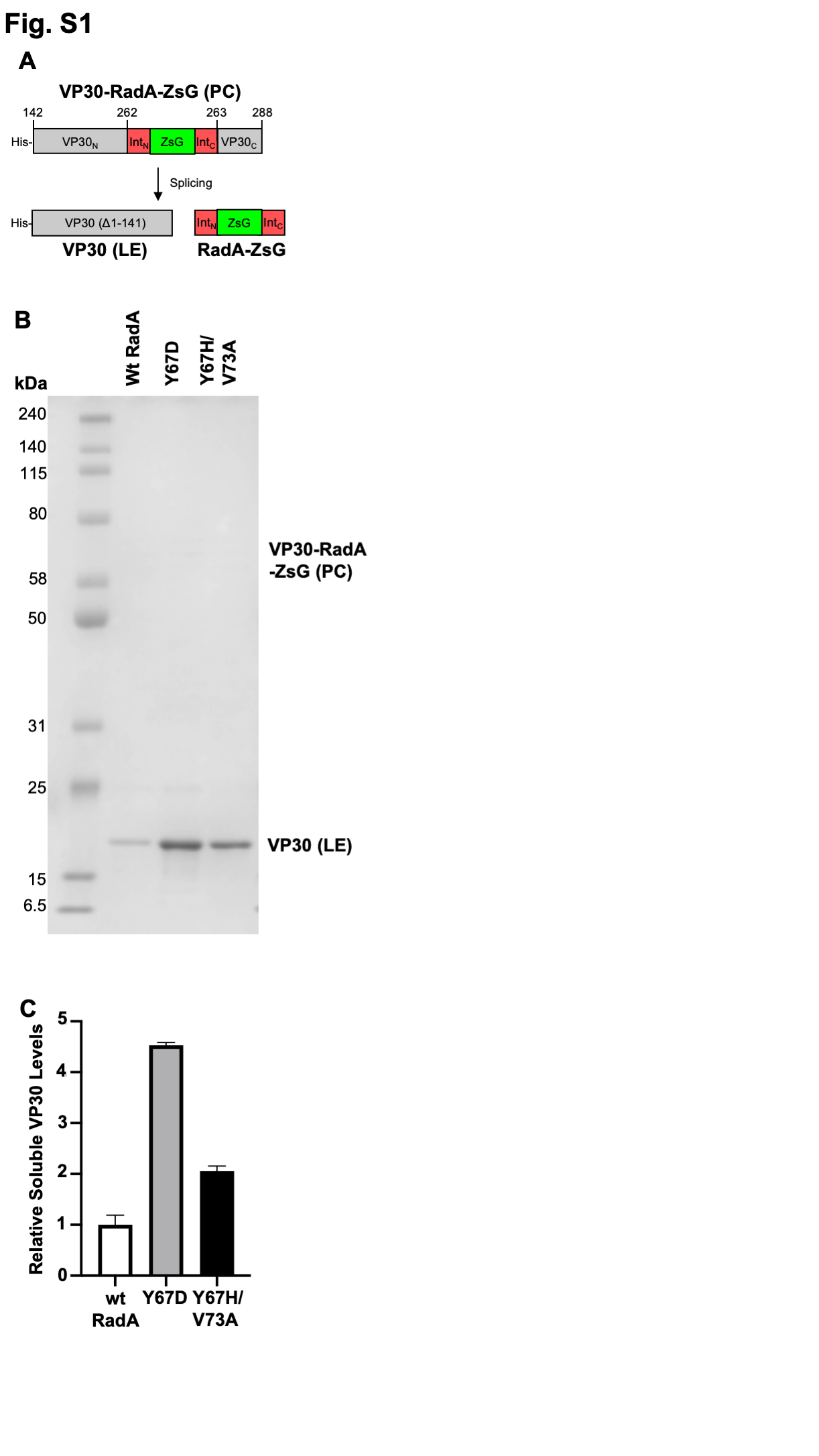

Supplement: Fig. S1 — Intein substitutions Y67D and Y67H/V73A improve intein activity within VP30-Int-ZsG context. [file mbio.00984-24-s0001.tiff]

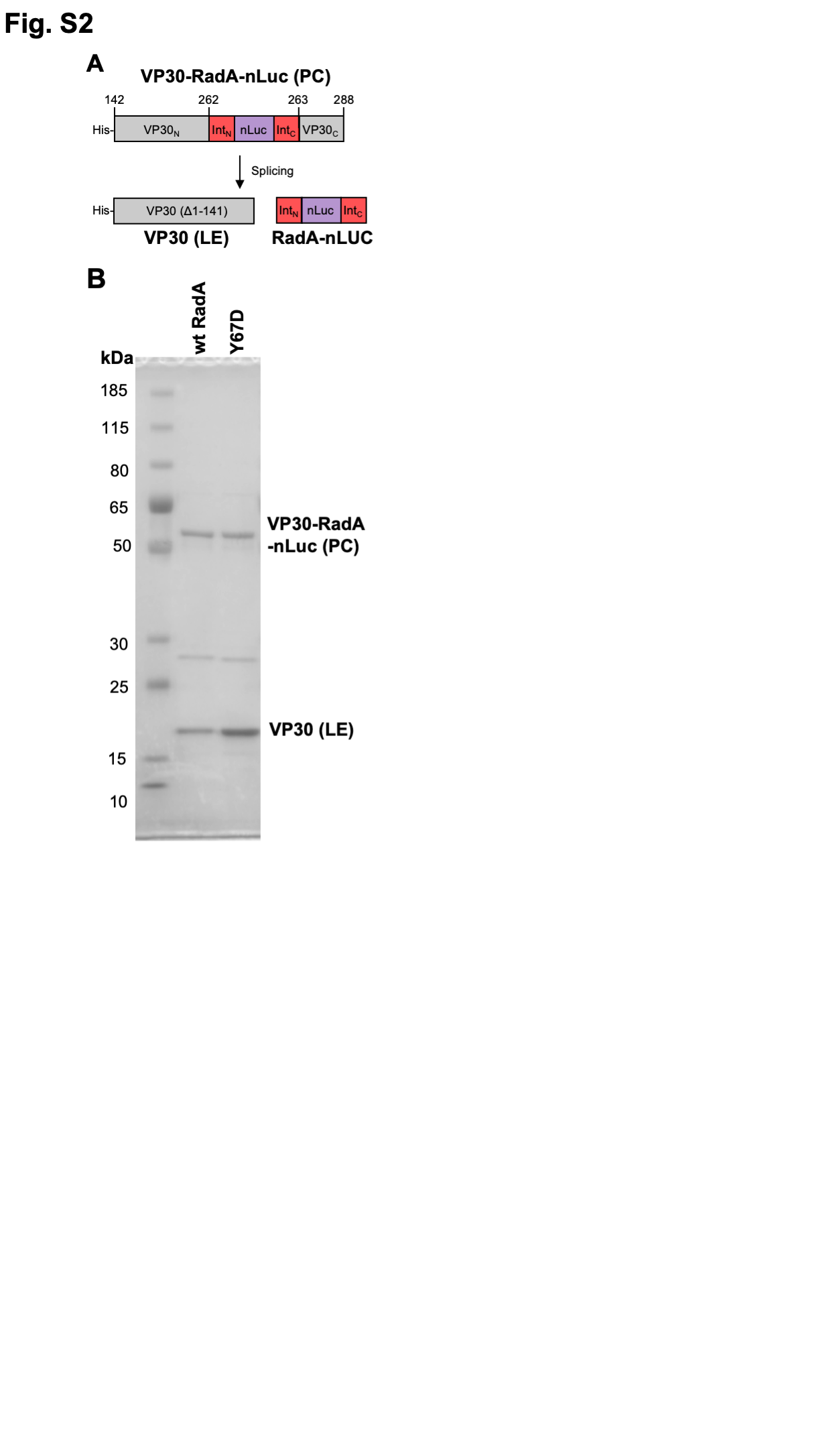

Supplement: Fig. S2 — Intein substitution Y67D improves intein activity within VP30-RadA-nLuc context. [file mbio.00984-24-s0002.tiff]

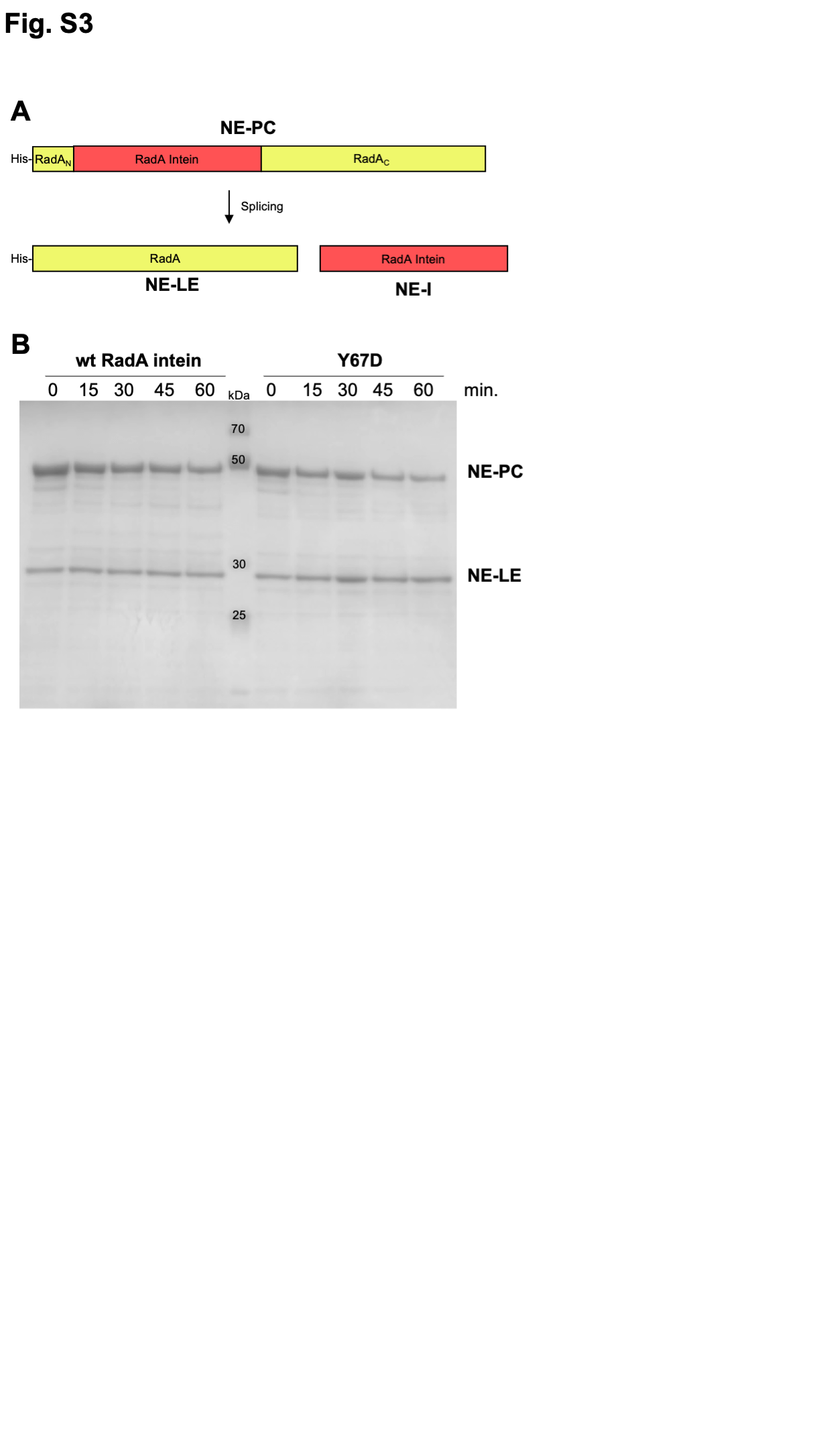

Supplement: Fig. S3 — Intein substitution Y67D improves intein activity within native RadA extein context. [file mbio.00984-24-s0003.tiff]
