# Supplementary material for: Improved protein splicing through viral passaging
Source: mBio. 2024 May 23;15(6):e00984-24. doi: 10.1128/mbio.00984-24 (PMC11237716; doi:10.1128/mbio.00984-24)
Supplement: Supplemental Information — Supplemental text, tables, and figures. [file mbio.00984-24-s0004.docx]

**Supplemental Information**

**Supplemental Figure 1. Intein substitutions Y67D and Y67H/V73A improve intein activity within VP30-Int-ZsG context. A.** Schematic of VP30 construct used in *E. coli*. The construct uses a truncated form of VP30 missing the first 141 amino acids with an N-terminal Histidine tag and RadA-ZsG (wild-type and mutant versions of RadA) inserted into VP30 between amino acids 262 and 263. **B.** SDS-PAGE following expression and quantitative pull-down of VP30-RadA-ZsG from the soluble fraction (wild-type and mutants). **C.** Quantification of VP30-RadA-ZsG relative levels of ligated exteins (LE) and precursor (PC) from panel A. Error bars represent standard deviation from three independent replicates. The expected sizes are 18.6 kDa for the LE and 64.5 kDa for the PC.

**Supplemental Figure 2. Intein substitution Y67D improves intein activity within VP30-RadA-nLuc context. A.** Schematic of VP30-RadA-nLuc reporter. **B.** SDS-PAGE following expression and quantitative pull-down of VP30-RadA-ZsG from the soluble fraction (wild-type and Y67D). The expected sizes are 18.6 kDa for the LE and 57.3 kDa for the PC. Quantification of results in triplicate are shown in Figure 2D of the main text. Error bars represent standard deviation from three independent replicates

**Supplemental Figure 3. Intein substitution Y67D improves intein activity within native RadA extein context. A.** Schematic of RadA native extein splicing construct. **B.** SDS-PAGE following incubation of purified RadA (wild-type and Y67D) at 55C for indicated times. T = 0 indicates level of spliced RadA following purification. The expected sizes are 29.3 kDa for the LE and 49.0 kDa for the PC. As previously observed (Lennon et al. 2019), the free intein is not resolved in this gel system. Quantification of results in triplicate are shown in Figure 2G of the main text. Error bars represent standard deviation from three independent replicates.

**Supplemental Table 1. Mutations found in passaged rEBOV-VP30-RadA-ZsG lineages.** Single nucleotide variable (SNV) tables of passage 2 (P2) rEBOV-VP30-RadA-ZsG as well as the three passage 13 rEBOV-VP30-RadA-ZsG lineages (P13A, P13B, and P13C), indicating the abundance and location within the genome of SNVs (SNVs ≥ 20% included in the Table). Frequency represents the abundance of the observed mutation, where a frequency of 1 equals 100% of the sequencing reads containing the described the mutation. Mutations within RadA are highlighted in yellow.

**MATERIAL AND METHODS**

*E. coli Plasmids and Protein Expression*

The plasmids used for protein expression in *E. coli* are shown in Table 1. In all cases except pΔN-RadAi, plasmids were synthesized, mutagenized (when applicable), and sequenced by Genscript. The construction of pΔN-RadAi was previously described (1).

| **PLASMID** | **DETAILS** |
| --- | --- |
| pVP30Δ1-141-RadA-ZsG (KanR) | EBOV VP30 Δ1-141 with RadA-ZsG inserted between VP30 residues 262 and 263 cloned into pET28 with an N-terminal His-tag |
| pVP30Δ1-141-RadA-ZsG Y67D (KanR) | pVP30Δ1-141-Pho-ZsG with intein codons67 mutagenized to aspartate |
| pVP30Δ1-141-RadA-ZsG Y67D/V73A (KanR) | pVP30Δ1-141-Pho-ZsG with intein codons 67 and 73 mutagenized to aspartate and alanine |
| pVP30Δ1-141-RadA-nLuc (KanR) | EBOV VP30 Δ1-141 with RadA-nLuc inserted between VP30 residues 262 and 263 cloned into pET28 with an N-terminal His-tag |
| pVP30Δ1-141-RadA-nLuc Y67D (KanR) | pVP30Δ1-141-Pho-nLuc with intein codon 67 mutagenized to aspartate |
| pΔN-RadAi (AmpR) | *P. horikoshii* RadA Δ1-115 cloned into pET45 with an N-terminal His-tag for NE-RadA expression |
| pΔN-RadAi Y67D (AmpR) | pΔN-RadAi with codon intein 67 mutagenized to aspartate |

***E. coli* expression plasmids used in this study.**

All plasmids were transformed into BL21(DE3) (New England Biolabs), cells were grown in LB broth with 50 μg/mL kanamycin or 100 μg/mL ampicillin to mid-log phase (OD_600_ 0.4-0.6), and protein expression was induced by the addition of 1 mM isopropyl β-D-1-thiogalactopyranoside (GoldBio). For quantitative His-tag pull-downs, fluorescence, and luminescence measuring with VP30 fusion constructs, proteins were expressed for 3 hr at 37°C. For purification of *P. horikoshii* RadA intein in the native exteins, proteins were expressed for 20 hr at 14°C.

*His-Tag Protein Pull-downs*

For pull-downs from the soluble fraction, following protein expression, cells were pelleted by centrifugation, resuspended in Ni^2+^ buffer A (20 mM Tris-HCl, 500 mM NaCl, 30 mM Imidazole, pH 8.0), sonicated by lysis, and insoluble materials were removed by centrifugation. For pull-downs from the whole cell fraction, cells were resuspended in 8M urea, sonicated by lysis, and insoluble materials were removed by centrifugation. Soluble lysates or the whole cell fractions were applied to Ni-charged MagBeads (Genscript), washed three times in Ni^2+^ buffer A, and eluted in Ni^2+^ buffer B (20 mM Tris-HCl, 500 mM NaCl, 300 mM Imidazole, pH 8.0). Between each binding, wash, or elution step, beads were collected using a magnetic rack.

To ensure quantitative comparison of total protein between samples, identical expression times, amounts of cells, Ni-charged MagBeads, and elution buffer were used.

*NE-RadA Purification and Splicing*

Following protein expression, cells were centrifuged, resuspended in Ni^2+^ buffer A, lysed by sonication, and subjected to a second round of centrifugation to remove insoluble materials from the clarified lysate. NE-RadA was purified using Ni-charged MagBeads by washing three times with Ni^2+^ buffer A, and eluted in Ni^2+^ buffer B. Dialfiltration was used to exchange NE-RadA into storage buffer (10% glycerol, 50 mM Tris, pH 8.0)

To initiate splicing, purified NE-RadA (1 mg/mL) was incubated at 55°C at indicated times in with 1 mM dithiothreitol (GoldBio). Time zero indicates the level of spliced NE-RadA following purification. NE-RadA wt and Y67D were purified under identical conditions concurrently. To stop splicing, samples were mixed with loading buffer (4X Bolt LDS; Invitrogen) and placed on ice.

*SDS-PAGE, Imaging, and Graphing*

Samples were combined with loading buffer (4X Bolt LDS; Invitrogen) and proteins were separated using Bis-Tris gels (8–16% Genscript) with MOPS buffer. Coomassie-stained gels were imaged using an Amersham Imager 680 (GE Healthcare) and bands were quantified by densitometry using ImageJ. Graphs were generated using Prism 9 (GraphPad) with the mean and standard deviation shown.

*Fluorescence and Luminescence Measurements*

Following expression of VP30 fusion proteins in *E. coli*, cells were pelleted by centrifugation, resuspended in Ni^2+^ Buffer A (20 mM Tris-HCl, 500 mM NaCl, 30 mM Imidazole, pH 8.0), and lysed by sonication. Following lysis, insoluble material was removed by centrifugation, and fluorescence or luminescence was measured in clarified lysates using a SpectraMax i3x microplate reader (Molecular Devices). For luminescence measurements, the Nano-Glo luciferase assay system (Promega) was used.

*Biosafety Statement*

All work with wild-type and recombinant Ebola virus (EBOV) was performed in the biosafety level 4 (BSL-4) facility of Boston University’s National Emerging Infectious Diseases Laboratories (NEIDL) following the approved standard operating procedures in compliance with local and national regulations pertaining to handling BSL-4 pathogens and Select Agents.

*Eukaryotic Cell Lines*

Eukaryotic cell lines used in this study include African green monkey kidney cells (Vero E6; ATCC CRL-1586) and human liver cells (Huh7; kindly provided by Apath L.L.C., New York, NY, USA). Vero E6 and Huh7 cells were maintained in Dulbecco’s modified Eagle medium (DMEM) supplemented with 10% fetal bovine serum (FBS), L-glutamine (200 mM), and either penicillin (50 units/mL) and streptomycin (50 mg/mL) or 100 ug/mL Primocin. All cell lines were grown at 37 °C/5% CO_2_.

*Viral Sequences*

The Zaire ebolavirus isolates, Ebola virus/H.sapiens-tc/COD/1976/Yambuku-Mayinga (NC_002549) NCBI reference filovirus sequence was used for cloning.

*Eukaryotic Expression Plasmids*

EBOV support plasmids (pCAGGS-NPEBOV, -VP35EBOV, -VP30EBOV, and -LEBOV) were described previously (2). A plasmid expressing a codon-optimized version of T7 RNA polymerase was previously described (3).

*Cloning of Full-Length EBOV Plasmids*

The full-length EBOV plasmid p15AK-EBOV (4), p15AK-EBOV-VP30-RadA-ZsG (p15AK-EBOV-VP30-ZsG-Int; 5), and pTwist-Amp-RadA-ZsG (pTwist-Amp-ZsG-Int; 5) were described previously. Plasmid p15AK-EBOV was kindly provided by H. Ebihara at the NIH NIAID Rocky Mountain laboratories. Point mutants within the RadA intein were generated by site-directed mutagenesis of the pTwist-Amp-ZsG-Int plasmid using the primers in Table 2. For creation of double mutants, the V73A mutant was first created and then was used as a template for the addition of the Y67D/Y67H mutants. Assembly of p15AK-EBOV-VP30-RadA-ZsG clones containing the RadA mutants was performed in the same manner as p15AK-EBOV-VP30-RadA-ZsG (5), replacing the pTwist-Amp-RadA-ZsG vector with sequence-verified point mutants.

| **RadA**  **mutant** | **Forward primer** | **Reverse primer** | **Annealing**  **temperature** |
| --- | --- | --- | --- |
| Y67D | AAGAGCTAGCGACATCTACAGAG | GTCTTCTTGATCTCGCC | 60°C |
| Y67H | AAGAGCTAGCCACATCTACAGAG | GTCTTCTTGATCTCGC | 57°C |
| V73A | CAGAGAGAAGGCGGAGAAGCTGA | TAGATGTAGCTAGCTCTTG | 60°C |

Primers used for site-directed mutagenesis in this study.

*Recombinant EBOV Rescue Transfection*

Recombinant EBOV rescue transfection was performed as described previously (3). Briefly, 1:1 mixtures of Huh7:Vero E6 cells (1 × 10^5^ cells plated per well of a 12-well plate) were transfected with EBOV support plasmids (125 ng pCAGGS-NP_EBOV_, 125 ng pCAGGS-VP35_EBOV_, 50 ng pCAGGS-VP30_EBOV_, and 500 ng pCAGGS-L_EBOV_), pCAGGS-T7 (20 ng; codon-optimized), and the recombinant EBOV-VP30-ZsG-Int full-length plasmid (1 μg) using TransIT-LT1 per manufacturer’s recommendations (Mirus Bio LLC). Media was changed approximately 18 h post-transfection and cells were monitored for cytopathic effect (CPE) and fluorescence for ZsGreen. Supernatants of cells showing CPE and fluorescence were transferred to T75 flasks of Vero E6 cells approximately 7–11 days post-transfection. Rescue of recombinant EBOV was performed in the BSL-4 facility of the NEIDL, following BSL-4 biosafety procedures.

*Virus Propagation*

EBOV Mayinga virus isolate was kindly provided by H. Feldmann at the NIH NIAID Rocky Mountain laboratories. Virus stocks were grown in Vero E6 cells, as previously described (6). Virus titers were determined in Vero E6 cells by 50% tissue culture infectious dose (TCID_50_) assay and calculated using the Spearman-Kärber algorithm. All work with EBOV was performed under BSL-4 conditions at the NEIDL, following approved SOPs.

*Viral RNA sequencing*

Total RNA from viral stocks was isolated using TRIzol-LS reagent (Invitrogen) according to the manufacturer’s protocol. RNA concentration was determined using a NanoDrop 1000 spectrophotometer (Thermo Fisher).

Sequencing libraries were prepared using the Illumina TruSeq v2 library kit without rRNA depletion or mRNA selection and were sequenced via Illumina NextSeq 2000. Raw FASTQ files were scanned for adventicious agents and other contaminants with Kraken2 v2.0.9 (7); none were detected. Reads were then aligned to the predicted viral genome using Bowtie2 v2.4.2 (8). Viral coverage was assessed with SAMtools v1.15 (9), and single nucleotide variants (SNVs) were called with LoFreq v2.1.3.1 (10). SNV annotation was performed with Biostrings v2.56.0 (11) and a custom R script.

Sequences of recombinant viruses determined by RNA-Seq have been uploaded to the NCBI under BioProject PRJNA1071083 (BioSamples SAMN39671338, SAMN39671339, SAMN39671340, and SAMN39671341), with individual sets of raw read data uploaded to the Sequence Read Archive: rEBOV-VP30-RadA-ZsG P2 (SRR27783919), rEBOV-VP30-RadA-ZsG P13A (SRR27783922), rEBOV-VP30-RadA-ZsG P13B (SRR27783921), and rEBOV-VP30-RadA-ZsG P13C (SRR27783920).

*Viral Replication Kinetics*

Viral replication kinetics were determined as described previously (3). Briefly, Huh7 cells (7 × 10^4^ cells plater per well of a 24-well plate) were infected with the indicated viruses at an initial MOI of 0.1. At the indicated times post-infection supernatant was clarified and virus titers were determined in Vero E6 cells by TCID_50_ assay and calculated using the Spearman-Kärber algorithm.

*Live-Cell Fluorescence Microscopy*

Cells infected with rEBOV-VP30-RadA-ZsG of different passages were imaged by live-cell fluorescence microscopy. Prior to imaging, cells were stained using Hoechst as follows; media was removed and replaced with PBS containing 40 µM Hoechst, cells were incubated at 37°C for 10 minutes and then washed 2x with PBS. Images were taken using a Nikon TS100 Eclipse microscope and Nikon DS Qi1Mc camera with NIS Elements F software.

**Supplemental References**

1. Lennon CW, Stanger M, Belfort M. 2016. Protein splicing of a recombinase intein induced by ssDNA and DNA damage. Genes Dev 30:2663–2668.
2. Schümann M, Gantke T, Mühlberger E. 2009. Ebola virus VP35 antagonizes PKR activity through its C-terminal interferon inhibitory domain. J Virol 83:8993–8997.
3. Hume AJ, Heiden B, Olejnik J, Suder EL, Ross S, Scoon WA, Bullitt E, Ericsson M, White MR, Turcinovic J, Thao TTN, Hekman RM, Kaserman JE, Huang J, Alysandratos K-D, Toth GE, Jakab F, Kotton DN, Wilson AA, Emili A, Thiel V, Connor JH, Kemenesi G, Cifuentes D, Mühlberger E. 2022. Recombinant Lloviu virus as a tool to study viral replication and host responses. PLoS Pathog 18:e1010268.
4. Tsuda Y, Hoenen T, Banadyga L, Weisend C, Ricklefs SM, Porcella SF, Ebihara H. 2015. An Improved Reverse Genetics System to Overcome Cell-Type-Dependent Ebola Virus Genome Plasticity. J Infect Dis 212 Suppl 2:S129-137.
5. Heiden B, Mühlberger E, Lennon CW, Hume AJ. 2022. Labeling Ebola Virus with a Self-Splicing Fluorescent Reporter. Microorganisms 10:2110.
6. Olejnik J, Forero A, Deflubé LR, Hume AJ, Manhart WA, Nishida A, Marzi A, Katze MG, Ebihara H, Rasmussen AL, Mühlberger E. 2017. Ebolaviruses Associated with Differential Pathogenicity Induce Distinct Host Responses in Human Macrophages. J Virol 91:e00179-17.
7. Wood DE, Lu J, Langmead B. 2019. Improved metagenomic analysis with Kraken 2. Genome Biol 20:257.
8. Langmead B, Salzberg SL. 2012. Fast gapped-read alignment with Bowtie 2. Nat Methods 9:357–359.
9. Li H, Handsaker B, Wysoker A, Fennell T, Ruan J, Homer N, Marth G, Abecasis G, Durbin R, 1000 Genome Project Data Processing Subgroup. 2009. The Sequence Alignment/Map format and SAMtools. Bioinformatics 25:2078–2079.
10. Wilm A, Aw PPK, Bertrand D, Yeo GHT, Ong SH, Wong CH, Khor CC, Petric R, Hibberd ML, Nagarajan N. 2012. LoFreq: a sequence-quality aware, ultra-sensitive variant caller for uncovering cell-population heterogeneity from high-throughput sequencing datasets. Nucleic Acids Res 40:11189–11201.
11. Pagès H, Aboyoun R, Gentleman R, DebRoy S. 2020 Biostrings: Efficient manipulation of biological strings. R package version 2560.
